# The Female Reproductive Tract Microbiota: Friends and Foe

**DOI:** 10.3390/life13061313

**Published:** 2023-06-01

**Authors:** Lokesh Kumar, Monika Dwivedi, Natasha Jain, Pranali Shete, Subhash Solanki, Rahul Gupta, Ashish Jain

**Affiliations:** 1 Genus Breeding India Pvt Ltd., Pune 411005, Maharashtra, Indiasubhashsolankhi2@gmail.com (S.S.); rahul.gupta@genusplc.com (R.G.); 2 Department of Pharmaceutical Sciences and Technology, Birla Institute of Technology, Mesra 835215, Jharkhand, India; 3 Department of Biotechnology, Chaudhary Charan Singh University, Meerut 250001, Uttar Pradesh, India; 4 Department of Microbiology, Smt. CHM College, University of Mumbai, Ulhasnagar 421003, Maharashtra, India; pranalikale2@gmail.com

**Keywords:** *Lactobacillus*, lactic acid, microbiota, probiotics, reproductive health, vagina

## Abstract

We do not seem to be the only owner of our body; it houses a large population of microorganisms. Through countless years of coevolution, microbes and hosts have developed complex relationships. In the past few years, the impact of microbial communities on their host has received significant attention. Advanced molecular sequencing techniques have revealed a remarkable diversity of the organ-specific microbiota populations, including in the reproductive tract. Currently, the goal of researchers has shifted to generate and perceive the molecular data of those hidden travelers of our body and harness them for the betterment of human health. Recently, microbial communities of the lower and upper reproductive tract and their correlation with the implication in reproductive health and disease have been extensively studied. Many intrinsic and extrinsic factors influences the female reproductive tract microbiota (FRTM) that directly affects the reproductive health. It is now believed that FRTM dominated by *Lactobacilli* may play an essential role in obstetric health beyond the woman’s intimate comfort and well-being. Women with altered microbiota may face numerous health-related issues. Altered microbiota can be manipulated and restored to their original shape to re-establish normal reproductive health. The aim of the present review is to summarize the FRTM functional aspects that influence reproductive health.

## 1. Introduction

The findings of Human Microbiome Project (HMP) proved the existence of a diverse microbial population and their eight million distinctive genetic elements throughout the human body, having elementary roles in human health and diseases [1]. It has been reported that about 30 trillion human cells/body, along with an estimated 39 trillion microbial cells, which includes bacteria, archaea, fungi, algae, and viruses, live on and inside the body [2,3]. Our “microbiota” comprises an assorted population of bacteria, viruses, fungi, and other unicellular organisms living in or on humans. The collection of all the genes within these microscopic organisms is known as the human “microbiome” [4]. The microbiome is not only the collection of genes, but also includes the structural elements, metabolites/signal molecules, and the surrounding environmental conditions [5]. Microbiomes have been studied intensively since the nineteenth century and are traditionally characterized using cultivation methods [1]. Recent findings have suggested the direct link of body microbiota in the regulation of various female reproductive complications such as endometriosis, PCOS (polycystic ovary syndrome), RPL (recurrent pregnancy loss), pregnancy complications, gynecologic cancer, and infertility [6,7,8]. Recent studies have also suggested that “vaginal seeding” (Wiping of infant’s body including mouth and face with its mother vaginal fluid) is helpful to restore the microbiome and the development of immunity, especially in the C-section delivery, where the newborn is devoid of direct exposure to the vaginal secretion of the mother [9]. Few studies consider the vaginal microbiome as a tool to predict the success of IVF/assisted reproductive technology [10]. An in-depth profile of the microbiome has recently been generated with the appearance of advanced molecular technology that demonstrated greater microbial diversity than previously recognized [11]. Interestingly, among the body’s microbiome, the specific female reproductive tract (FRT) houses nine percent of the total microbial population of the entire body [12]. Most investigations have been focused to study the microbiota of the lower reproductive tract (LRT) [13]. However, recent investigations proved the presence of a diverse microbial ecology in the endometrium and other locations of the upper reproductive tract (URT) [14,15]. The microbial burden is progressively reduced from reproductive tract’s lower to upper portion [16,17]. The composition of LRT microbiota changes during the entire female’s lifecycle from childhood to reproductive age and up to menopause [18]. Hormonal changes in a woman are one of the critical factors that regulates the microbiota configuration at different stages of a woman’s life [19]. The cervicovaginal microbiota is extensively screened and categorized into at least six types, named community state types (CSTs) [13,20]. Human females have *Lactobacillus* spp. as the predominant group in the pool of FRTM, while in the other mammals, the *Lactobacillus* population is merely more than 1% [21,22]. Lactic acid, the predominant metabolic byproduct of *Lactobacillus* when glycogen serves as the primary substrate, resulted in an exceptionally low pH (≤4.5) in the lower reproductive tract [23]. Certain Community State Types (CSTs) dominated by *Lactobacillus* spp., principally *L. crispatus*, are more correlated to reproductive eubiosis than CSTs having less abundant *Lactobacilli* [13]. The optimum composition of the FRTM, dominated by *Lactobacillus* spp. and acidic pH, diversely benefits the host. Several external and host-associated factors may disturb the optimum composition of normal microbiota, which leads to compromised reproductive health and severe gynecological conditions, including BV, sterility, and preterm delivery, and are a more significant threat of sexually transmitted infections (STIs) [24]. Many strategies have been projected to effectively restore optimum balance in the FRTM, including antibiotics, probiotics, hormone replacement therapy (HRT), vaginal fluid transplant, and a combination of any two or more approaches [25,26]. The purpose of the present review is to summarize the existing information on the FRTM, its role in reproductive health, and the future direction of FRTM analysis.

## 2. Microbiota of Female Reproductive Tract

Distinct microbial communities exist throughout the female reproductive tract (FRT), starting from the vaginal opening to the placenta [27,28]. The lower reproductive tract (LRT) comprises the vagina and cervix together, known as the cervicovagina. In most recent studies, cervicovaginal microbiota are generally studied together [29]. The cervicovaginal microbiota resides in and on the epithelium’s outermost layer. In the LRT, a healthy cervicovagina demonstrates the dominancy of *Lactobacillus* spp. (10^7^–10^9^ *Lactobacilli*/gram of vaginal fluid) that accounts for up to 95% load of the total bacterial population residing in the entire RT [30,31]. The cervicovaginal microbiota of reproductive-aged females has been categorized into five major clusters, termed community state types (CSTs). Out of five, four CSTs exhibited dominancy of *Lactobacillus* spp. CST-I is dominated by *L. crispatus*, whereas CST-II, CST-III, and CST-V show dominancy of *L. gasseri*, *L. iners*, and *L. jensenii*, respectively. The fifth one, CST-IV, has a lower density of *Lactobacillus* spp. [13]. CTS-IV is categorized into two subgroups, A and B. Subgroup IV-A comprises a modest population of *Lactobacillus* spp. and other species, i.e., *A. vaginae, G. vaginalis*, and *Prevotella* spp. Subgroup IV-B comprises microbial species including *A. vaginae*, *Leptotrichia* spp., and *Mobiluncus* spp. [20,27]. Interestingly, it has been observed a shifting of different CST populations in different parts of the reproductive tracts of women [32] (Table 1).

The upper reproductive tract (URT) comprises the endocervix, endometrium, uterine cavity, fallopian tubes, ovary, peritoneal fluid, and placenta. The existence of bacteria in the URT remains controversial and for a long time has been considered a germ-free region. Recent studies have challenged this “sterile womb” dogma by proving the colonization of bacteria in the URT even in the absence of any infection [11,16]. The origination of microbiota identified in the URT is still unclear. It is hypothesized that they ascend from the vagina probably due to spontaneous uterine contractions, which are most intense during ovulation and orgasms [33]. Bacterial load gradually decreases from the LRT to the URT. Uterine bacteria were estimated to be about 10,000 times lesser than that of the cervicovagina, and the most dominant ones were *Prevotella* spp., *L. iners*, and *L. crispatus* [16].

**Table 1:** Comparative description of different Community State Types (CSTs) on the basis of prominent organism, pH, Nugent score, pregnancy status, major cell type, and reproductive health.

Community State Type	Prominent Organism (% Dominancy) [13]	Median pH (All Ethnic Groups) [13]	Nugent Score [13,27]	%Nonpregnant Women [34]	%Normal Pregnancy [34]	Epithelial Cells [35]	Reproductive Health
**CST-I**	*L. crispatus* (26.2%)	4.0 ± 0.3 (Lowest pH)	Lowest Nugent score (0–3)	17	38.1	Mature squamous cells (MSCs)	Healthy condition
**CST-II**	*L. gasseri* (6.3%)	5.0 ± 0.7	Nugent score (4–6)	8.9	4.3	MSCs	Healthy condition
**CST-III**	*L. iners* (34.1%)	4.4 ± 0.6	Low Nugent score (0–3)	35.2	51.8	MSCs/# Immature parabasal cells	Healthy condition (less stable or more in transition) [36]
**CST-IVA**	No particular prevailing species Different levels of *L. inners* or other *Lactobacillus* spp., with low proportions of *Anaerococcus*, *Corynebacterium Finegoldia*, *Streptococcus* [27]	5.3 ± 0.6 (highest pH) CST-IVB has higher pH than CST-IVA	Relatively lower Nugent scores than IV-B (7–10)	10.4	3.6	MSCs/# Immature parabasal cells	Risk associated with PTB and obstetrical complications [34,37] Associated with HPV infection, CIN, and HIV acquisition [38] Dominant in postpartum stage [39] CST-IV are risk factors for BV [32]
**CST-IVB**	No particular prevailing species Comparatively high levels of *Atopobium*, *Gardnerella*, *Mobiluncus*, *Peptoniphilus*, *Sneathia*, *Prevotella*, and several other taxa of BVAB [20,27]		Contains some of the BV-associated bacteria (BVAB) and is often associated with highest Nugent scores (7–10)	28.5	2.2		
**CST-V**	*L. jensenii* (5.3%)	4.7 ± 0.4	Nugent score (4–6)			MSCs	Healthy condition

# Desquamative inflammatory vaginosis.

Additional groups steadily recognized were *Bifidobacterium*, *Corynebacterium*, *Staphylococcus*, and *Streptococcus* [40]. *Lactobacillus* is the most dominant group that constantly exists in the URT. Endometrial fluid may be broadly categorized into two clusters: (i) the *Lactobacillus*-dominated (LD) cluster and (ii) non-*Lactobacillus*-dominated (NLD) clusters. Aagaard et al. proposed that the placenta is a house of metabolically active and less-abundant microbiota that are composed mainly of nonpathogens of the Bacteroidetes, Proteobacteria, Firmicutes, Fusobacteria, and Tenericutes phyla [41]. The microbiota of healthy female fallopian tubes has yet to be well characterized. Pelzer et al. identified *Enterococcus* sp. and *Staphylococcus* sp., However, *Lactobacillus* sp. is the most abundant microflora present in a fallopian tube, along with other sp., including *Pseudomonads*, *Propionibacterium*, and *Prevotella* [28]. Recently Chen et al. identified a variety of microbiomes as a signature, primarily of *Facklamia*, *Erysipelothrix*, and *Pseudomonas* in the fallopian tube and *Morganella*, *Pseudomonas*, *Sphingobium*, and *Vagococcus* in peritoneal fluid [16].

## 3. Factors That Influence the Composition of FRTM

**Figure 1:**
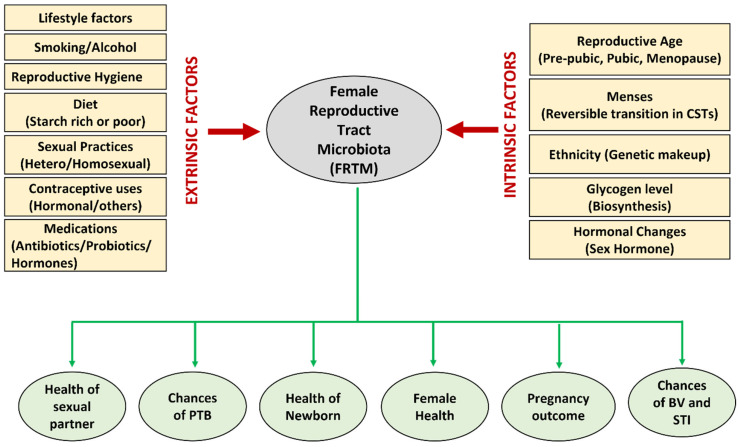
Various extrinsic and intrinsic factors that influence the composition of the FRTM, and various aspects of reproductive health directly or indirectly affected by the microbiota.

**Figure 2:**
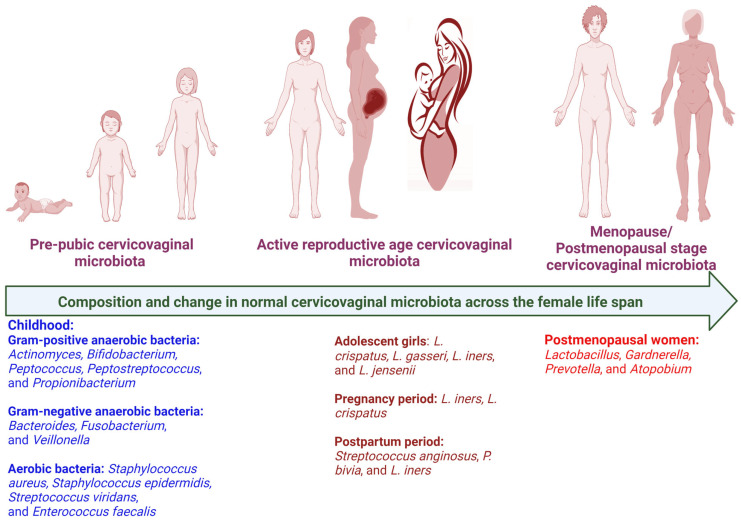
Composition and change in cervicovaginal microbiota in healthy individuals across the female life span [52]. Created with BioRender.com (accessed on 15 May 2023).

Several endogenous and environmental factors directly influence and alter the FRTM composition and cervicovaginal milieu (Figure 1). A starch-rich diet increases glycogen levels in the vagina, thus creating a favorable environment to proliferate *lactobacilli* [21]. The prepubic cervicovaginal microbiota are rated as relatively stable build-ups of aerobes, anaerobes, and intestinal microbial communities, which primarily shows the dominancy of anaerobes, i.e., the *Enterobacteriaceae* and/or *Staphylococcacee* family [42]. In the active reproductive age, due to the elevated level of estrogen, lactic acid bacteria colonize the vagina, which contributes to the acidification of the cervicovaginal region by discharging principally lactic acid and some other organic acids [43]. The dominancy of *Lactobacillus* is maintained throughout the reproductive phase. During the menopausal stage, the estrogen level drops, a thinner vaginal epithelium containing low glycogen and reduced mucin secretion results in a less dominant *Lactobacillus* population, and hence an elevated vaginal pH (>5), rendering the female genitourinary tract more susceptible to infections [44]. In pregnant women, the absence of menses, an increased level of sex hormones (placental estrogen), and a thicker vaginal mucosa stuffed with glycogen leads to increased glycogen metabolism and reduced pH (<4.5) [45] (Figure 2). The low vaginal pH, due to lactic acid production, may contribute to the lower bacterial diversity and greater dominancy of *Lactobacillus* sp., hence reducing the risk of BV and other infections during pregnancy [34]. 

It has been reported that different races or ethnic groups have different microbial compositions due to the diversity in their genetic constitution [46]. Sexual behavior and the lifestyle of the host are the leading factors that influence the FRTM. Homosexual relationships, unprotected sex, and having multiple, new, or numerous male partners negatively affect vaginal homeostasis [47,48]. Additionally, reproductive hygiene, the type of contraception, and antibiotic treatments also have directly influenced the FRTM. It has been also reported that detergent-based nonspecific vaginal contraceptives can also adversely affect normal microbiota of reproductive tract [49]. Hormonal contraceptives can stimulate the colonization of beneficial *lactobacilli* and are supposed to have a role in the stabilization of balanced vaginal microbiota and reduced risk of BV [50]. It is observed that broad-spectrum antimicrobials can adversely affect the harmful bacteria as well as reduce the number of beneficial bacteria in the RT [51].

## 4. *Lactobacillus*: The Key to Female Reproductive Health

**Figure 3:**
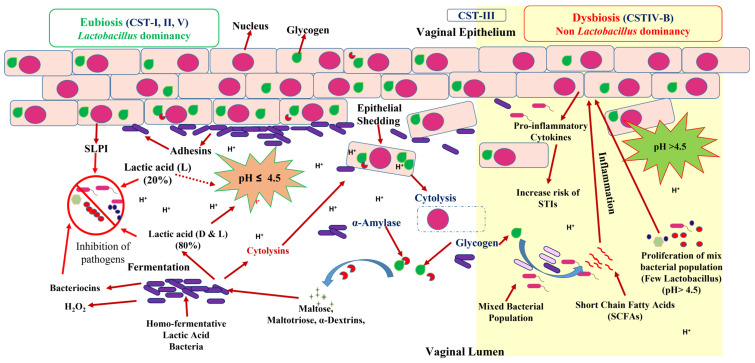
Complex mechanism and factors responsible for vaginal eubiosis and dysbiosis. Eubiosis: Dominancy of *Lactobacillus* spp., production of lactic acid, H_2_O_2_, bacteriocins, adhesins, SLPI, biofilm, and consistent acidic pH in vaginal lumen. Dysbiosis: Non-*Lactobacillus* spp. dominancy, SCFAs and mixed acid production, increased pH, proinflammatory cytokines production, inflammation, and epithelial shedding in the vaginal lumen.

**Figure 4:**
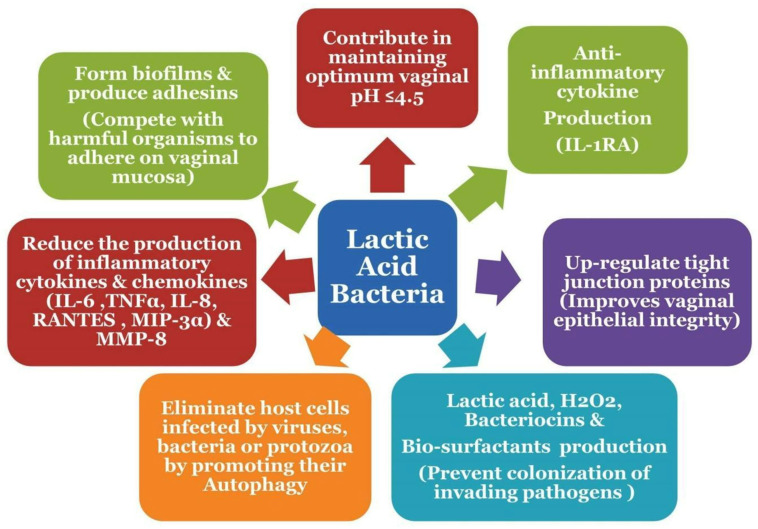
Distinct roles of lactic acid bacteria and their metabolites in maintaining reproductive health.

Lactic acid is the crucial factor for vaginal homeostasis, majorly (80%) produced by *Lactobacillus* spp. and in minor amounts (20%) by vaginal epithelial cells [53]. A healthy vaginal microbiota of a reproductive-aged woman is usually dominated by Gram-positive, facultatively anaerobic, catalase-negative, rod-shaped, nonsporulating bacteria of *Lactobacillus* spp. [54]. In reproductive age, elevated levels of estrogen in premenopausal women induce glycogen accumulation in the vaginal epithelium. Hormonal changes induce continual shedding of these glycogen-rich cells in the vaginal lumen. Upon cytolysis, released glycogen catabolizes into maltose, maltotriose, and α-dextrins by the host’s α-amylases, which are further fermented into lactic acid by the action of lactate dehydrogenase (LDH) of the *Lactobacillus* [55]. Lactic acid acidifies the cervicovaginal mucosa by maintaining an acidic pH (≤4.5) (Figure 3). Several *Lactobacilli* spp. also produce hydrogen peroxide (H_2_O_2_), biosurfactants, and proteinaceous bacteriocins, which synergize with lactic acid and prevent the colonization of invading pathogens. However lactic acid, not H_2_O_2_, is the main antimicrobial element in the reproductive tract (RT) synthesized by *Lactobacillus* spp. [56]. Lactic acid also exerts an anti-inflammatory effect in the RT by stimulating anti-inflammatory cytokine IL-1RA production and reducing the proinflammatory cytokine and chemokine (interleukin-6, tumor necrosis factor α, interleukin-8, MIP-3α, and RANTES) production [57]. A healthy microbiota is considered to be an endogenous guard of the female reproductive tract. *Lactobacillus* species adhere to the vaginal mucosa and compete with harmful organisms, thus preventing the colonization of pathogens on the vaginal epithelium. *Lactobacillus* upregulates tight junction proteins, thus improving epithelial integrity, forming biofilms and modulating the expression of cytokines and receptors by the host cells. Moreover, they eliminate the infected cells, mainly by stimulating autophagy (Figure 3) [58,59]. The low vaginal pH and high viscosity of vaginal mucous and the *Lactobacillus*-mediated inhibition of bacterial adhesion on the cervicovaginal lining are the main elements that favor the dominancy of *Lactobacillus* spp. [60]. In any circumstances, if *Lactobacillus* dominancy is lost, diverse bacterial species occupy the vaginal epithelium and stimulate the production of inflammatory signaling molecules responsible for the employment of immune cells and inflammation. This diverse bacterial population also reduces the viscosity of the cervicovaginal fluid (CVF) by the action of mucin-degrading enzymes [61]. Mucus barrier degradation and depletion may be a crucial parameter in the etiology of BV and the adverse health outcomes linked with it [62] (Figure 4). However, it is also reported that some females can maintain cervicovaginal eubiosis in a non-*Lactobacillus*-dominant community; in such cases, lactic acid is produced by the microorganisms of *Atopobium*, *Megasphaera*, *Leptotrichia*, *Staphylococcus*, and *Streptococcus genus* [63]. *Lactobacillus* spp. are capable of synthesizing D(−) and L(+) optical isomers of lactic acid, whereas vaginal cells produce only the L(+) isomer [64,65]. D(−) isomer reduces the level of matrix metalloproteinase-8 (MMP-8) synthesis [66]. MMP-8 can degrade the cervical plug, thus facilitating the entry of microorganisms in the URT [67]. Hence, a higher level of D(−) lactic acid in the cervicovaginal environment can positively affect the reproductive health of pregnant women by preventing UTR infections. *Gardnerella vaginalis* and *L. iners* generally found in BV are poor D(−) lactic acid producers. Hence, D(−) lactic-acid-producing *L. crispatus*-dominant CSTs are more associated with female reproductive health compared to poor D(−) lactic acid producers such as *L. iners* [68].

## 5. Effect of FRTM on Female Fertility and Sperm Function

Different microbial species of the FRTM can modulate conception, pregnancy, childbirth, and outcomes of infertility treatment [69]. In the FRT, the presence of *Enterococci*, *Enterobacteriaceae*, *Streptococci*, *Staphylococci*, and Gram-negative bacteria are responsible for increased miscarriage risk and reduced chances of implantation [13]. A reduced endometrial *Lactobacillus* population is evident among in vitro fertilization (IVF) patients (38%) versus healthy women (85.7%), which indicates the alteration of the FRTM may be associated with infertility [70]. A recent study revealed that the FRTM could directly influence the IVF success rate and reported that the IVF success rate was 9% in dysbiotic women patients, while in eubiotics, it was 44%. [71]. This study also reported that microbiota evaluation of the FRT could also be an important biomarker to assess the reproductive status of women.

Spermatozoa are viewed as foreign bodies by the FRT. Hence, there is always a risk of antibody production against spermatozoa that can reduce fertility [72]. Vaginal microbiota dominated by *Lactobacillus* spp. act to diminish the chances of the development of antisperm immunity [73,74]. *Escherichia coli* is a habitually isolated organism in genital infections reported to adversely affect sperm motility [73]. Fimbriae of *E. coli* interacts with the surface receptors of sperm, which leads to its association with sperm and their agglutination [75]. Findings of some in vitro studies suggest the effect of genital tract infections on sperm motility reduction is mediated by induced sperm membrane lipid peroxidation [13]. Immune cells attracted by genital tract infections can generate reactive oxygen species (ROS) and inflammatory cytokines, which adversely affect the sperm physiology in the FRT [76]. ROS-mediated membrane lipid peroxidation is associated with reduced sperm movement [77]. Soluble products of *Lactobacillus* spp. could protect sperm cells from oxidative damage, preserving spermatozoa’s motility and vitality [78]. Recently reports have also been demonstrating the adverse effects of some *lactobacilli* on sperm movement, which may also function as a biological filter for a combination of unhealthy sperm with eggs [79].

## 6. Preterm Birth and FRTM

Any birth not before twenty weeks, but before thirty-seven completed weeks of gestation, is defined as a preterm birth (PTB) [80]. Genitourinary tract inflammation caused by BV or reproductive tract infections could be a possible factor of PTB [81]. The ascent of microorganisms from the cervicovagina to the uterus, placenta, and fetal membranes may account for 25–40% of PTBs [34]. Preterm premature rupture of membranes (PPROM) is strongly correlated with the altered FRTM in distinct studies. Pregnant females with symptoms of PPROM rarely have microbiota dominated by *Lactobacillus* spp. and show a diverse cervicovaginal bacterial population [82]. A previous study reported that a low population of *L. crispatus* and more BV-associated bacterium-1 (BVAB1), *Prevotella* cluster 2, *Sneathia amnii*, *Gardnerella*, Ureaplasma, *Megasphaera* type 1, and BVAB-TM7 have a high probability of PTM compared to controls [83]. In addition, the risk of PTB may also be correlated with a strong association of *Mobiluncus curtsii/mulieris* and *Sneathia sanguinegens*, *Atopobium*, *M. curtsii/mulieris*, and *Megasphaera.* Interestingly, the risk of PTB is low in women having a high population of *Lactobacillus* in the genitourinary tract [29]. It is well documented that antibiotics which reduced the risk of maternal infection may not reduce the occurrence of PTB [84]. These antibiotics could exhibit a toxic effect on these pathogens and detrimental effects on the advantageous FRTM. 

## 7. FRTM and Endometriosis

Endometriosis is a chronic uterine gynecological disorder characterized by the growth of endometrium tissue outside of the uterine cavity, commonly on the peritoneal cavity. Several studies have reported the pathogenesis of endometriosis, including immunologic abnormalities, endometrial disorders, and peritoneal dysfunction, that could also be linked with uterine carcinogenesis and infertility [8,85]. The FRTM plays an important role in endometriosis. Previous clinical studies demonstrate that certain microbes, such as *Corynebacterium*, *Enterobactericaea*, *Flavobacterium*, *Pseudomonas*, and *Streptococcus*, are found to dominate in endometriosis patients compared to controls [86]. Chang et al. described that the composition of the FRTM in endometriosis patients is different from healthy women. They also observed the distinct microbiome in Stage I and II as compared to those in Stages III and IV in endometriosis patients compared to the healthy ones [87]. However, more in-depth research is needed to establish the direct association between the treatment strategies of endometriosis patients and disease biomarkers using the FRTM. 

## 8. FRTM and Gynecological Cancer

The microbiome of the gastrointestinal and female reproductive systems is thought to impact carcinogenesis and responsiveness to anticancer treatment. Any alterations within FRTM may result in the development and progression of malignancies complications including gynecologic cancer. It has been reported that dysbiosis could itself favor a procarcinogenic state through alterations in the host immune response, hormone homeostasis, and alternations in the cell cycle and apoptosis [7]. The reduction in cellular barrier protection and chronic modification of the local immune response could be caused, by which the cervical and vaginal microbiota influences the risk of cervical dysplasia and the development of invasive cervical cancer. *Gardnerella vaginalis* may facilitate viral infection inducing a proinflammatory state and damage to the barrier of the cervical mucous [88]. Certain other microbiota, such as *Staphylococcus*, *Blautia*, *Parabacteroides*, *Atopobium vaginae*, and *Prophyromonas* spp., could cause DNA damage and apoptosis by producing toxic metabolites and the generation of reactive oxygen species, while also upregulating the oncogenic pathways and proinflammatory cytokines [7]. In contrast, *Lactobacillus* spp. has been shown to promote the tumor-suppressive environment in the female reproductive tract by producing metabolites, anti-inflammatory cytokines, and the downregulation of oncogenic pathways.

## 9. FRTM in Relation to Vaginal Eubiosis and Dysbiosis

Cervicovaginal eubiosis is characterized by the dominancy of the *Lactobacillus* genus, which maintains a healthy environment through lactic acid production [24]. Displacement of *Lactobacillus*-dominant optimal vaginal microbiota by diverse bacterial populations has been associated with multiple gynecological complications broadly known as vaginal “dysbiosis” [89]. The most frequent type of cervicovaginal dysbiosis is BV, which is a polymicrobial clinical syndrome of reproductive-aged women, characterized by the massive reduction and displacement of the *Lactobacillus* population by other anaerobic and facultative bacteria, diversity in the vaginal microbiota, production of amino compounds, and an elevated vaginal pH (>4.5) [90]. Bacterial-vaginosis-associated bacteria (BVAB) increase vaginal pH by utilizing available lactic acid for metabolism and producing acetic acid, propionic acid, butyric acid, isobutyric acid, succinic acid, formic acid, fumaric acid, and additional short-chain fatty acids (SCFAs). SCFAs raise the release of proinflammatory cytokines from cervicovaginal epithelial cells, which results in a higher risk of acquiring STIs [91]. Most of the organisms associated with BV are also members of the endogenous normal vaginal microbiota. It is believed that BV could enhance the risk of STIs such as human papillomavirus (HPV), human immunodeficiency virus (HIV), *Trichomonas vaginalis*, *C. trachomatis*, and *Neisseria gonorrhoeae* [92]. Hence, BV should not be considered as an STI. BV is typically associated with an elevation in the level of proinflammatory cytokines and increases the vaginal pH by reducing the level of an antimicrobial peptide “secretory leukocyte protease inhibitor” (SLPI) [93], thus enabling the proliferation of acid-sensitive nonendogenous infectious organisms. BV has been associated with complications in pregnancy, adverse effects on newborns, chorioamnionitis, premature deliveries, pelvic inflammatory disease (PID), fetal loss, cuff cellulitis, postpartum endometritis, cervicitis, and an increased risk of genitourinary infections [94].

Previous studies have reported that BV is associated with approximately 1.5 times higher chances of HIV infection [95,96]. The presence of abnormal microbiota in the cervicovaginal region can cause a strong inflammation, with massive recruitment of CCR5^+^ CD4^+^ T-lymphocytes and a raised titer of IL-1β, IL-17, IL-23, and other inflammatory cytokines, thus increasing susceptibility of HIV infection. These immune cells also display a triggered phenotype (HLA-DR+CD38^+^) and show acute susceptibility to viral multiplication. Females with a diverse RTM had seventeen times more active CD4^+^ lymphocytes than the females with *Lactobacillus dominancy* [97]. Women with abnormal microbiota have fewer cervical gamma delta 1 (GD1) cells, which have a defensive role against HIV [98]. Studies suggested lactic acid and low vaginal pH can inactivate HIV [99].

## 10. FRTM in Relation to STIs

**Human papillomavirus (HPV)** is a cluster of viruses and is the most common STI. There are more than one hundred types of HPV, of which at least fourteen can cause a malignant growth known as high-risk (HR)-type HPV. Noncancer-causing HPV is grouped in the low-risk (LR)-type HPV [100]. HR-HPV is believed to be the main factor responsible for the progression of cervical cancer, including the cancer of other genital organs in women and men. HPV-16 and HPV-18 are accountable for 70% of cervical cancers. Increased CVM diversity is associated with HR-HPV infection [101]. A higher abundance of non-*Lactobacillus* spp. or *L. iners* was associated with 3–5-fold greater risk of HPV and a 2–3-fold greater risk for HR-HPV and cervical malignancy compared to when *L. crispatus* was the dominant organism [102].

**Trichomoniasis**, caused by the extracellular protozoan parasite *Trichomonas vaginalis*, is the most widely recognized nonviral, sexually transferred infection worldwide. *T. vaginalis* and *lactobacilli* contend for a grip on the vaginal epithelium [103]. With certain exceptions, *Lactobacillus* inhibits *T. vaginalis* from adhering to the cervicovaginal epithelium in a species-specific or strain-specific manner [104]. Previous studies have shown that trichomonas infection could increase by several fold the incidence of HIV, including other STIs, i.e., gonorrhea, human papillomavirus (HPV), and herpes simplex virus (HSV) [105]. It has been demonstrated that *Lactobacillus gasseri* of the vaginal environment creates a physical barrier and uses pharmacological-type processes to counteract the detrimental cytotoxic effects of *T. vaginalis* [106].

***Candida*** species are designated as an opportunistic pathogen of the FRT and are considered the main factors (85–95% occurrence) associated with vulvovaginal candidiasis (VVC) patients; it is considered the second most prevailing dysbiosis after BV [107]. Studies suggested that lactic acid bacteria inhibit the *Candida* yeast-to-hyphae switch, and by competing with it for adhering to epithelial receptors, keep up its low number in the RTMB. Moreover, an acidic pH and the antimicrobial components of *Lactobacillus* origin suppress its overgrowth and transition from avirulent to virulent hyphal form [108].

## 11. Strategies to Restore the FRTM to Improve Reproductive Health

Modulating and re-establishing a healthy FRTM could potentially improve women’s reproductive health [109]. Restoration of lactic-acid-producing bacteria in the FRT could improve the reproductive health of patients with abnormal microbiota [110]. To deal with this issue, different strategies are under consideration, including antibiotics, probiotic formulation, hormone replacement therapy (HRT), and vaginal fluid/microbiome transplantation. Broad-spectrum antibiotics used for vaginal pathogens could impair not only the growth of targeted pathogens but also the off-target flora of different body parts [32,111]. Other drawbacks of antimicrobial drugs are drug resistance, higher probability of recurrent infections, and many consequential adverse outcomes due to the depletion of the endogenous off-target microbiota of other organs [112]. The use of probiotics (living–beneficial and nonpathogenic microorganisms) is also another accepting strategy to modulate the reproductive tract by replacing abnormal flora and for selecting normal microbiota through intermittent doses of the probiotic formulation. This approach restores healthy microbiota without any adverse effects on the bodily physiology [113].

Several probiotic formulations are under trial to treat BV, VVC, and other forms of dysbiosis. A few probiotics claimed to promote cervicovaginal health with promising outcomes are summed up in Table 2, which may be the future of probiotic therapy to treat RT infections and restore eubiotic conditions without any side effects often associated with antibiotic treatments. In a two-step treatment, pathogenic bacteria are first targeted and eliminated by antimicrobial compounds, and in the second step, the cervicovagina is populated with beneficial *lactobacilli* using suitable probiotic formulations [114]. Women who received HRT restored the *Lactobacillus* population in the vagina [25]. Some potential side effects reported with HRT include vaginal bleeding, perineal pain, and breast pain [115]. Recent studies explain that vaginal fluid transplantation from a healthy donor to a dysbiotic recipient could restore the normal microbiota in the FRT and help in the re-establishment of vaginal eubiosis [111].

## 12. Conclusions and Future Directions

It is well established that human microbiota, “the forgotten organ”, is not an invader but a beneficial colonizer. The FRTM maintains a healthy environment by dominating infectious microorganisms and is accountable for the normal functioning of the entire reproductive system. An abnormal and more diverse microbiota can adversely affect reproductive health. Today, different types of microbial communities and their relative quantity are known due to the advent of new sequencing techniques; however, to address the entire complexity of the whole microbial population of the reproductive tract, much detailed investigation is needed. Many aspects of FRTM are yet to be answered:

- Does every individual species of microbiota have an advantageous function or not?
- Why do the *lactobacilli* predominate, specifically in humans and not in other mammals?
- What is the role of the host genetic composition in shaping the microbiota?
- Is there any contribution of a mother’s cervicovaginal microbiota in establishing her infant microbiome?
- Does the mother’s microbiota affect the reproductive, obstetric, and overall health consequences of the progeny?
- Despite an enormous number of *Lactobacillus* spp., why are only a few of them dominant?
- Is there any cooperation between the microbiota of the reproductive tract and of other body parts, or vice versa?

The dominance of *Lactobacillus* spp. raises the question of the role of other ignored microscopic organisms that coexist with them. The role of each single member of the FRTM should be explored irrespective of their ratio, which have been neglected in previous investigations. New in-vitro and in-vivo experimental models and vaginal chips should be developed and the influence of their microbiome should be investigated on the overall health of the experimental model. The FRTM is significantly influenced by a variety of environmental and lifestyle factors; in addition, the influence of host genetics in shaping the host microbiome is also anticipated. However, it is challenging to distinguish between the genetic and environmental influences and the effect is currently smaller than the first estimations. Despite the undeniable significance of reproductive tract microbiota, little is known about their molecular mechanism in reproductive health. There is a considerable challenge to explore the detailed pathway of complex microbiota which influence numerous aspects of female reproductive health. However, it is well established that the mother’s microbiota affects the reproductive health, obstetric, and progeny outcome, but detailed study is needed to evaluate the influence of each member of the FRTM on various aspects of female health, pregnancy and obstetric results, and the long-term health of both mother and infant. Future research on the FRTM should be focused on developing diagnostic tools based on using these microbiotas as biomarkers of a specific physiological or clinical status, as well as new approaches that explore entirely the variety and functionality of the microbiome and its relations with the host. Analysts should design a stable, balanced, effective, universal, and safe formulation of microbiota that can be used to restore the FRTM irrespective of ethnicity and demographic differences. Metagenomics and contemporary sequencing technologies have enabled the identification of a significant number of bacterial species that were earlier inaccessible by culture-based approaches. Metatranscriptomics, metaproteomics, and community metabolomics should be used to supplement sequencing results. New bioinformatics tools should be developed and used for the processing and analysis of a massive amount of sequence data. Future studies should concentrate on examining the intricate dynamics and interactions between various FRTM members and how they affect and are affected by the remaining human microbiome. Future research should be focused on opening the way to novel opportunities for the betterment of female reproductive health.

## Figures and Tables

**Table 2:** Some promising probiotic formulations, their microbial components, and the outcome of probiotic therapy developed to treat BV and VVC and to maintain reproductive health.

Probiotic Formulation	Bacterial Strain	Outcome of Probiotic Therapy
Gynophilus [116]	*L. casei rhamnosus* Lcr35	After clindamycin treatment, 7 days of Gynophilus was significantly efficacious in curing BV; 69/83 cured
Florisia [117]	*L. brevis* CD2 + *L. salivarius* subsp. *salicinius* FV2 + *L. plantarum* FV9	7 days of use without any antibiotic significantly cured BV (15/18 Nugent 0–3)
RC-14/GR-1 [118]	*L. fermentum* RC-14 + *L. rhamnosus* GR1	5 days of treatment without any antibiotic was significantly more efficacious in curing BV than 5 days of metronidazole gel
Unnamed [119]	*L. delbrueckii* subsp. *lactis* DM8909	7 days of *L. delbrueckii* without any antibiotic had the same potency as metronidazole gel in treating BV
Physioflor [120]	*L. crispatus* IP 174178	14 days of use after metronidazole treatment, +14 days in three subsequent menstrual cycles, significantly reduced BV; 16/39 cured
*L.acidophilus* LA14 [121]	*L. acidophilus* LA14	14 days of treatment without any antibiotic significantly reduced BV cases, 46/60 cured, and VVC, 9/60 cured
Kramegin [122]	*L. acidophilus* + *Krameria triandra* plant extract + 15 mg lactic acid	10 days of treatment without any antifungal cured 75/75 cases of acute VVC, and 20/30 cases of recurrent VVC
ActiCand [123]	*L. fermentum* LF10 *+ L. acidophilus* LA02	Without any antifungal; significantly cured VVC cases, 7/30 cured
Estromineral Probiogel [124]	*L. fermentum* LF10 + *L. plantarum* LP02	Without any antifungal, cured 51/82 patients of acute VVC and 27/27 cases of recurrent VVC
Gelatin–oil–probiotic suppository [125]	*Bacillus coagulans* Unique IS-2	Decrease in the cfu of *Candida* in infected rat
Multi-strain probiotic formulation [126]	*Lactiplantibacillus plantarum* PBS067, *Lacticaseibacillus rhamnosus* LRH020, and *Bifidobacterium animalis* subsp. *lactis* BL050	*T. vaginalis* is completely inhibited, and the growth of *C. glabrata* and *N. gonorrheae* is decreased

## Data Availability

Not applicable.
